# Latency-Optimal Computational Offloading Strategy for Sensitive Tasks in Smart Homes

**DOI:** 10.3390/s21072347

**Published:** 2021-03-28

**Authors:** Yanyan Wang, Lin Wang, Ruijuan Zheng, Xuhui Zhao, Muhua Liu

**Affiliations:** School of Information Engineering, Henan University of Science and Technology, Luoyang 471023, China; wangyanyan@stu.haust.edu.cn (Y.W.); linwang@haust.edu.cn (L.W.); zxh@haust.edu.cn (X.Z.); lxk0379@126.com (M.L.)

**Keywords:** back-pressure algorithm, computational offloading, edge cloud computing, lyapunov drift, smart home

## Abstract

In smart homes, the computational offloading technology of edge cloud computing (ECC) can effectively deal with the large amount of computation generated by smart devices. In this paper, we propose a computational offloading strategy for minimizing delay based on the back-pressure algorithm (BMDCO) to get the offloading decision and the number of tasks that can be offloaded. Specifically, we first construct a system with multiple local smart device task queues and multiple edge processor task queues. Then, we formulate an offloading strategy to minimize the queue length of tasks in each time slot by minimizing the Lyapunov drift optimization problem, so as to realize the stability of queues and improve the offloading performance. In addition, we give a theoretical analysis on the stability of the BMDCO algorithm by deducing the upper bound of all queues in this system. The simulation results show the stability of the proposed algorithm, and demonstrate that the BMDCO algorithm is superior to other alternatives. Compared with other algorithms, this algorithm can effectively reduce the computation delay.

## 1. Introduction

Nowadays, with the popularity of Internet of Everything (IoE) applications [1], smart homes [2] have become more and more intelligent and convenient, which has also promoted the rapid growth of new mobile applications with high latency requirements, such as intelligent lighting control systems and so on. These mobile applications typically require real-time responsiveness and a lot of computing resources. It is no doubt that this results in higher requirements for smart devices in smart homes. However, due to the constraints of the base hardware and their physical size, the computing resources of smart devices are generally limited [3] and cannot meet the requirements of these applications. Therefore, the technology of edge cloud computing (ECC) [4,5,6] is considered an effective and promising way to handle the challenges between the smart devices with limited resources and the mobile application with high demand. Different from the traditional cloud computing [7,8], ECC is more suitable for dealing with the sensitive tasks with low latency. It can distribute the management and calculation of the services in a smart home, which can greatly improve the operating efficiency.

In recent years, the offloading problem in an ECC system [9,10] has attracted much attention. Computational offloading migrates tasks from the local device to the edge cloud for computing, typically from devices with limited computing resources to resource-rich cloud processors [11]. Moreover, the single-user terminal multi-edge cloud processor offloading frame has gradually been unable to meet the rapid increase of smart devices. As a result, a lot of research on multi-user devices and multi-edge processors has emerged. An online algorithm for joint radio and resource management in the multi-user edge cloud was proposed in [12] to achieve the goal of minimizing the power consumption of local devices and cloud servers. Chen et al. [13] studied a multi-user computing offloading scheme in the case of multi-channel wireless interference in the edge cloud system.

In the computational offloading of multiple smart devices and multiple edge processors, there are many algorithms on how to obtain the offloading decision-making of task [14,15]. An effective offloading method to minimize the computational delay of tasks was proposed [16]. A transmission power scheduling method was presented in [17] by optimizing the energy consumption of tasks. However, they all ignore that there may be a large number of tasks waiting to be processed on the local smart device or edge cloud processor, which cannot meet the low latency requirements of a large number of sensitive tasks in the system. In addition, some studies have considered the waiting delay. Li et al. [18] considered the waiting delay and proposed a computational offloading game to save computing resources and response time. Geng et al. [19] studied a computational offloading strategy, which also took into account the waiting delay. Meng et al. [20] shown an offloading method based on Markov decision process (MDP). But none of them specifically optimize the delay from the perspective of optimizing the task backlog.

To solve the above problems, we consider the combination of the stochastic optimization method [21] and the back-pressure algorithm [22] in a busy queue system where sensitive tasks arrive randomly. The system includes multiple local smart device queues and multiple edge processor queues. The sensitive tasks are those tasks that have deadlines and low latency requirements. We used the Lyapunov drift optimization theory [21] to minimize the queue length of tasks in each time slot. This method can realize the stability computation at each time interval, and optimize the computing delay while ensuring the stability of the system. In addition, we propose an offloading algorithm for sensitive tasks, termed back-pressure algorithm-based computational offloading strategy for minimizing delay (BMDCO), to obtain the offloading decision of tasks and the number of tasks that can be offloaded. The BMDCO algorithm optimizes the computational delay of sensitive tasks by taking into account not only the delay of tasks, but also the backlog of task queues. The major contributions of this paper are summarized as follows:

- We constructed a system including local queues and edge queues and define the Lyapunov drift optimization problem to minimize the average queue length in each time slot, which ensures the stability of all queues.
- We present the back-pressure algorithm-based computational offloading strategy for minimizing delay, which can determine the task offloading decision and offloading number by computing the task delay and using the back-pressure algorithm, while also being subject to the time allowance of the task.
- We provide the theoretical analysis of the BMDCO algorithm stability, and the simulation results are given to show the stability of the BMDCO algorithm and demonstrate that the performance of this algorithm outperforms other comparison alternatives.

The rest of the content is organized as follows. Section 2 describes the related work and Section 3 introduces the system model, the queue dynamics, and the problem formulation. In Section 4, we introduce and analyze the details and performance of the BMDCO algorithm. The numerical results of this strategy are given in Section 5, and Section 6 presents the conclusion of this paper.

## 2. Related Work

Recently, how to design an efficient offloading scheme is still a challenging research problem. In recent years, a great deal of research work has emerged on the offloading of edge cloud computing [23,24,25]. Moreover, Sundar et al. [26] proposed an individual time allocation heuristic algorithm based on a greedy algorithm to obtain the offloading decision of each task and minimize the cost. Zhang et al. [27] studied an energy-delay optimization service model. Similarly, Mao et al. [28] and Kuang et al. [29] studied a single-user edge cloud computing system with multiple tasks, with the goal of minimizing the trade-off between latency and energy consumption by combining optimal scheduling, offloading decisions, and transmission power allocation. Due to the rapid increase of smart devices and higher application requirements, the offloading frame of multi-user device multi-edge processor [30,31,32] has gradually replaced the mode of single-user device multi-edge processor. Guo et al. [30] studied the offloading strategy of collaborative computing between cloud and edge cloud under the framework of hybrid fiber-wireless access network. Chen et al. [31] modeled the offloading problem of computing tasks for a multi-user multi-task system in mobile edge cloud, and obtained the offloading decision of tasks by using the Lyapunov theory. A method of resource allocation in ECC was studied [32], which used the stochastic optimization technology to minimize the cost and improve the capability of the server at the same time.

However, a large number of current offloading strategies are designed by minimizing computing delay or energy consumption. The authors of [33] developed a dynamic offloading and resource scheduling scheme with efficient energy consumption, which can reduce the energy consumption of tasks, shorten the computing delay of tasks, and satisfy the requirement of task dependency. To solve the low delay requirement of tasks, Liu et al. [34] presented a new system design that used the extreme value theory to reduce the power consumption and balance the allocated resources. An iterative heuristic algorithm was presented in [35] to reduce the computational delay problem of the tasks in the system. Zhang et al. [36] researched an energy-aware task offloading method to jointly optimize resource allocation and communication in the case of delay sensitivity. Liu et al. [37] studied an offloading method weighing energy consumption and execution delay in mobile cloud computing with the assistance of cloud computing.

Among the research work mentioned above, some did not consider the task backlog, and some just converted the task backlog into the computational delay of the task. There has been no research on offloading methods specifically considered from the perspective of the task backlog. In this paper, we consider a system containing multiple local smart device queues and multiple edge cloud processor queues and use the stochastic optimization method. By minimizing the Lyapunov drift optimization problem, the queue backlog of tasks in each time slot is minimized, while ensuring the stability of all queues.

So far, there has been some research works [38,39,40] about the stochastic optimization technique and the Lyapunov method. In order to minimize the computation cost of tasks, Meng et al. [20] have shown an offloading method based on the Markov decision process (MDP). This method used the queues but only considered the cascading relationships between queues. Chen et al. [41] studied an online offloading framework for peer nodes in a small cellular network based on Lyapunov technology, so as to achieve the objective of minimize the energy consumption of the network and maximize the network performance. A method of allocating cloud resources considering user demands was proposed [42], and modeled user requirements used the stochastic optimization method, which ensured the uncertainty of cloud requirements and minimized the total cost. An offloading method based on the Lyapunov optimization theory was presented in the edge cloud system [43] to get the offloading decision. Merluzzi et al. [44] studied a dynamic offloading algorithm for joint optimization of radio and computing resources in a task queue system. This algorithm was based on stochastic optimization technique to obtain the offloading decisions and ensure a certain excess probability.

The above-mentioned literature all use the queue theory and verify that the stochastic optimization method can reach good performance; however, they still need to improve in the aspect of delay optimization. In this paper, we combine the Lyapunov drift with the back-pressure algorithm to jointly optimize the computational delay of the task. The back-pressure algorithm can drive the offloading of tasks on the local queue through calculating the task backlog difference between the local smart device queue and the edge processor queue, which can effectively reduce the delay. But it is currently mostly used in network routing, and there is little research on edge computing [45]. We apply the back-pressure algorithm to the computational offloading in ECC for the first time. Therefore, we propose a computational offloading strategy for minimizing delay based on back-pressure algorithm, which can obtain the offloading decision and the number of offloading tasks, and reduce the computing delay of tasks.

## 3. System Model and Problem Formulation

In this section, we focus on the detailed analysis of the proposed queue system model in the paper. It mainly includes two parts: network model and queue dynamics. Besides, we also construct the definition of the optimization problem of this queue system.

### 3.1. System Model

**Figure 1:**
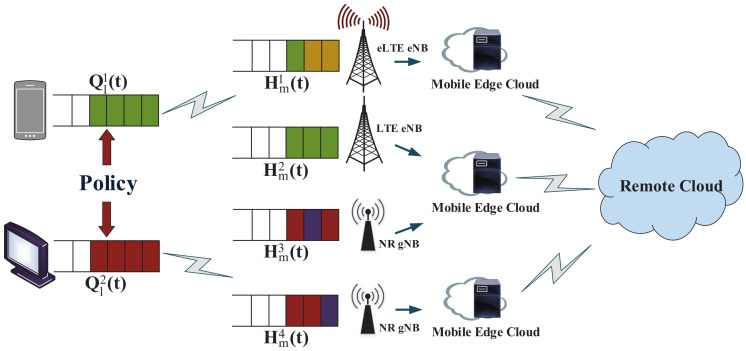
The system model diagram.

In this paper, we consider a queue system with random busy task arrivals containing multiple smart devices and multiple edge processors. The system model considered in this paper is shown in Figure 1. To better describe the model, we assume that the local smart devices of the system are all connected to alternating current (AC). The local smart devices refer to the user devices with different execution speeds that have many sensitive tasks, require high latency, and have limited computing power. For convenience, we also refer to local smart devices as local devices or smart devices. The base station *m* associated with the edge processor in edge cloud computing system is denoted as the set M. Three types of base stations are considered here, namely LTE eNB, eLTE eNB, and NR gNB, which have different processing rates. And we assume that the remote cloud has infinite computing power, which can execute multiple tasks at once. The computing delay of the task in the remote cloud is negligible.

#### 3.1.1. Local Smart Device and Tasks

Let L be the set of *L* local smart devices. The computing tasks are identified as *N* task nodes, denoted as N. In this paper, the tasks on the smart device can be executed locally named local computing or offloading from the smart device to the edge processor for execution called edge computing. We assume that the transmit power of the local smart device is fixed, and the computing tasks on the device cannot be divided into subtasks for execution. We consider that the task *i* on the smart device must be completed before its deadline $F_{i}$. In this system, we define the time *t* as the slot with duration τ, and let T be the set of *T* indexes of the time slots.

In this paper, we define the task offloading decision for each task *i* to indicate where the task is executed. Denote the task offloading decision variable as $y_{lm}^{i}\left(t\right)\in \left\{0,1\right\}$, where t∈T,i∈N,l∈L,m∈M. Specifically, if $y_{lm}^{i}\left(t\right)=0$, the task *i* will be computed on the local smart device *l* in slot *t*, and otherwise, the task *i* will be offloaded to the gNB *m* for execution. Note that one task cannot be executed simultaneously on both local device and edge processor.

#### 3.1.2. Local Computing Model

For the first case, the task on the local device is computed locally. We represent $f_{l}^{i}$ as the CPU cycle frequency of the device when task *i* is executed on the local smart device *l*. According to dynamic voltage and frequency scaling techniques (DVFS), we can change the computing rate of the local device by adjusting the CPU cycle frequency [46]. Hence, we define the local computing rate of the task *i* in the time slot *t* as

(1) $$v_{l}^{i}\left(t\right)=\alpha \left(t\right)f_{l}^{i},$$

where α(t) is the scaling factor between the packet size of a task and the floating point computation. Assuming that the tasks in this queue system all have the same size, we define the number of CPU cycles require to compute each task as $f_{i}$. Then the local computing delay of the task *i* is

(2) $$t_{l}^{i}=\frac{f_{i}}{v_{l}^{i}\left(t\right)}.$$

Notice that since we assume that the device in this system are connected to AC, energy consumption is temporarily not considered in this paper.

#### 3.1.3. Edge Computing Model

In the second case, the task is offloaded to the edge cloud for computation. It includes two processes: the transmission process on the offloading link from the local smart device to the edge processor and the computing process on the edge processor in edge cloud.

First, we define $h_{lm}^{i}\left(t\right)$ as the channel gain in time slot *t* that the task *i* is offloaded from the local smart device *l* to the edge processor *m*, and $\rho_{i}\left(t\right)$ is the transmission power in slot *t* of the task *i*. The transmission rate of the task *i* offloaded from the local device *l* to the edge processor *m* in time slot *t* can be represented as

(3) $$v_{lm}^{i}\left(t\right)=\omega \log_{2}\left(1+\frac{\rho_{i}\left(t\right)h_{lm}^{i}\left(t\right)}{\sigma }\right),$$

where ω is the channel bandwidth and σ is the additive white Gaussian noise (AWGN) power of the channel used by task *i*. We further denote the maximum rate of channel transmission in the system as $v_{lm}^{max}\left(t\right)$. Moreover, since the task size is the same, we define the amount of data for each task as $D_{i}$. Then, the transmission delay for the task *i* is

(4) $$t_{lm}^{i}=\frac{D_{i}}{v_{lm}^{i}\left(t\right)}.$$

In addition, we denote $f_{m}^{i}$ as the computation capability of the edge processor *m*. The computing rate of task *i* on the edge processor *m* in slot *t* is

(5) $$v_{m}^{i}\left(t\right)=\alpha \left(t\right)f_{m}^{i}.$$

Then, the computational delay of offloaded task *i* on the edge processor *m* is

(6) $$t_{m}^{i}=\frac{f_{i}}{v_{m}^{i}\left(t\right)}.$$

### 3.2. Queue Dynamics

In this paper, we define that the tasks on both the local smart device and the edge processor are in the queue. Figure 1 shows that the queue system model for computational offloading. Assuming that the smart device and the edge processor can only execute one task at a time, and other tasks are waiting in their respective queues in this model. Then use the offloading algorithm to determine whether the computing task is executed locally or offloaded to the edge cloud to improve the performance of user. A detailed description of the queue construction in the model is given below.

Let $Q_{l}^{i}\left(t\right)\in \left[0,\infty \right)$ and $H_{m}^{i}\left(t\right)\in \left[0,\infty \right)$ be the queues of task *i* on the local device *l* and edge processor *m* in time slot *t*, respectively, which are used to store the tasks that the device or processor needs to compute. Define $A_{l}^{i}\left(t\right)$ as the randomly arriving tasks to be executed on the local device *l* in slot *t*. We assume that it is independent and identically distributed (i.i.d) in every time slot, with mean $E\left[A_{l}^{i}\left(t\right)\right]=\lambda_{l}^{i}$, where $\lambda_{l}^{i}$ is the average arrival rate of task *i* on the local device *l*. As a consequence, the dynamics of the task queue on the local device in adjacent time slots is given by

(7) $$Q_{l}^{i}\left(t+1\right)={\left[Q_{l}^{i}\left(t\right)-v_{l}^{i}\left(t\right)-\sum_{m=1}^{M}y_{lm}^{i}\left(t\right)v_{lm}^{i}\left(t\right)\right]}^{+}+A_{l}^{i}\left(t\right),$$

where ${\left[\cdot \right]}^{+}=\max\left\{\cdot ,0\right\}$ and the first term of Equation (7) represents the remaining unexecuted tasks in the local device queue currently. This term is defined as the length of the local queue minus the sum of tasks that can be computed locally and tasks that will be offloaded to the edge cloud.

In addition, we express the dynamics of the queue on the each edge processor as

(8) $$H_{m}^{i}\left(t+1\right)={\left[H_{m}^{i}\left(t\right)-v_{m}^{i}\left(t\right)\right]}^{+}+\sum_{l=1}^{L}y_{lm}^{i}\left(t\right)v_{lm}^{i}\left(t\right)+A_{m}^{i}\left(t\right),$$

where $A_{m}^{i}\left(t\right)$ is denoted as the randomly arriving tasks on the edge processor in slot *t*, with mean $E\left[A_{m}^{i}\left(t\right)\right]=\lambda_{m}^{i}$. The first term of Equation (8) is the uncomputed tasks on edge processor queue in slot *t*. The second term represents the task of offloading from the local smart device to the edge processor. The sum of the last two items shows all newly arrived tasks on the edge cloud in the slot *t*.

There is a coupling relationship between the local device queue dynamics in Equation (7) and the edge processor queue in Equation (8), that is, the departure of the task on the local device queue is the arrival of one of the edge processor queues. In order to make the system more realistic, we define the newly arrived tasks $A_{m}^{i}\left(t\right)$ on the edge cloud to represent the tasks to be processed by the edge processor itself. Due to the high frequency of the processor, we think it will not affect this relationship. The relationship can also be expressed equally in the following equation, which is defined as:

(9) $$Q_{tot}^{i}\left(t\right)=Q_{l}^{i}\left(t\right)+H_{m}^{i}\left(t\right),$$

in which $Q_{tot}^{i}\left(t\right)$ signifies the total number of the tasks in slot t. What this means is that the total number of tasks in time slot *t* is equal to the sum of the tasks on the local device and the edge processor.

### 3.3. Problem Formulation

In this section, we define the optimization problem of the queue model. In the first place, we construct a quadratic Lyapunov function about queues $Q_{l}^{i}\left(t\right)$ and $H_{m}^{i}\left(t\right)$ according to the Lyapunov optimization theory, which combines the queues on all local smart devices and the queues on each edge processors in the system. Denote the Lyapunov function as follows:

(10) $$V\left(t\right)=\frac{1}{2}\sum_{l=1}^{L}\sum_{i=1}^{N}{\left[Q_{l}^{i}\left(t\right)\right]}^{2}+\frac{1}{2}\sum_{m=1}^{M}\sum_{i=1}^{N}{\left[H_{m}^{i}\left(t\right)\right]}^{2},$$

where Equation (10) is a strictly increasing function. Then we define the Lyapunov drift function as

(11) Δ(t)=E[V(t+1)−V(t)|Z(t)],

where $Z\left(t\right)=(Q_{l}^{i}\left(t\right);H_{m}^{i}\left(t\right))$ is a vector of the queues on the local devices and the edge processors in time slot *t*. By minimizing Equation (11), we can minimize the queue backlog of tasks in each slot, while ensuring the stability of all queues. Therefore, we give the definition of Lyapunov drift optimization problem, which can be expressed as

(12) $$\begin{array}{cc} \max_{y_{lm}^{i}\left(t\right)} & \sum_{l=1}^{L}\sum_{i=1}^{N}Q_{l}^{i}\left(t\right)\left(v_{l}^{i}\left(t\right)+\sum_{m=1}^{M}y_{lm}^{i}\left(t\right)v_{lm}^{i}\left(t\right)-A_{l}^{i}\left(t\right)\right)\\ \;\;\; & +\sum_{m=1}^{M}\sum_{i=1}^{N}H_{m}^{i}\left(t\right)\left(v_{m}^{i}\left(t\right)-\sum_{l=1}^{L}y_{lm}^{i}\left(t\right)v_{lm}^{i}\left(t\right)-A_{m}^{i}\left(t\right)\right), \end{array}$$

subject to

$$\begin{array}{cc} \; & \left(a\right)\;y_{lm}^{i}\left(t\right)\in \left\{0,1\right\};\\ \; & \left(b\right)\;Q_{tot}^{i}\left(t\right)\leq Q_{max}\left(t\right);\\ \; & \left(c\right)\;v_{lm}^{i}\left(t\right)\leq v_{lm}^{max}\left(t\right);\\ \; & \left(d\right)\;t_{l}^{i},t_{lm}^{i}+t_{m}^{i}\leq F_{i}; \end{array}$$

in which $Q_{max}\left(t\right)$ is the maximum number of tasks that a queue can accept. The meanings of these constraints are as follows: Constraint (a) ensures that the offloading decision variable for the task is either 0 or 1. (b) guarantees that the total number of tasks in a queue over slot *t* does not exceed the maximum number accepted by each queue in the queue system. (c) indicates that the transmission rate of the task cannot surpass the maximum channel transmission rate of this system. In the end, constraint (d) represents that whether the task is executed locally or processed in the edge cloud, its completion time must be within its deadline.

Equation (12) is derived by minimizing the Lyapunov drift Δ(t). We can minimize the average queue length of tasks in each time slot *t* by solving the optimization problem, while achieving queue stability. The derivation process of the drift optimization problem (12) is shown in Appendix A. In order to obtain the offloading decision and the number of offloaded for the sensitive tasks, we propose a computational offloading strategy based on back-pressure algorithm for minimize the delay under the condition of ensuring the stability of all queues and minimizing the average queue backlog.

## 4. Back-Pressure Algorithm-Based Offloading Strategy of Minimizing Delay

The proposed strategy is based on minimizing the computational delay of tasks. It is mainly aimed at sensitive tasks with deadlines in the smart home. In order to better describe this algorithm, we first describe its main steps in detail in the first section. Next, we further illustrate the performance of this proposed algorithm and give the theoretical analysis.

### 4.1. Algorithm Development

In this queue system, the offloading decision of the task *i* must meet its deadline $F_{i}$. To prevent the system from offloading all tasks to the edge cloud for execution to meet the task completion deadline, the strategy takes into account not only the computational delay for the execution of the task, but also the queue backlog. To this end, we propose the BMDCO algorithm. The details of this algorithm are shown in Algorithm 1, and the major steps of the algorithm are shown as follows:

#### 4.1.1. Possible Offloading Task Set

For the tasks that have deadlines in the queue system, our objective was acquire the appropriate decision to minimize the computational delay of it. In this step, we separately computed the delay when the task is executed locally or offloaded to the edge cloud processing; Then, we compared the results. If the execution delay of the task on the edge cloud is less than that on the local smart device, we put it into the set *S*, where *S* is the set of tasks that may be offloaded.

**Algorithm 1** BMDCO Algorithm
 1:**Input:***N*, *L*, *M*, τ, *t*, *T*, $D_{i}$, $f_{i}$, $F_{i}$, $Q_{l}^{i}\left(0\right)$, $H_{m}^{i}\left(0\right)$. 2:**Output:** Task offloading decision {$y_{lm}^{i}\left(t\right)$} and task offloading number {$y_{lm}^{i}\left(t\right)v_{lm}^{i}\left(t\right)\tau$}. 3:Initialize target queue system; 4:**while***t* = 0 **to** *T* **do** 5: **for** *i* = 1 **to** *N* **do** 6:  Compute $t_{l}^{i}$ from (2); 7:  Compute $t_{lm}^{i}+t_{m}^{i}$ from (4), (6); 8:  **if** $t_{lm}^{i}+t_{m}^{i}<t_{l}^{i}$ **then**
 9:   i∈S; 10:  **else** 11:   $y_{lm}^{i}\left(t\right)=0$ {Execute *i* on the local device}; 12:  **end if** 13:  **for** i∈S **do**
 14:   Compute $W_{d}^{i}\left(t\right)$ from (14); 15:   Find $i^{*}$ from (15); 16:   **for** $i^{*}\in S^{*}$ **do**
 17:    $y_{lm}^{i^{*}}\left(t\right)=1$ {Execute $i^{*}$ on the edge cloud}; 18:    Obtain the task offloading number; 19:   **end for** 20:  **end for** 21:  **if** $\left(t_{l}^{i}+wait_{l}^{i}\left(t\right)\right)>F_{i}∥\left(t_{lm}^{i}+t_{m}^{i}+wait_{m}^{i}\left(t\right)\right)>F_{i}$ **then**
 22:   Execute the fallback option; 23:   **return** 24:
**  end if**
 25: **end for** 26: Update queues $Q_{l}^{i}\left(t\right)$ and $H_{m}^{i}\left(t\right)$ according to (7) and (8) in each time slot, respectively. 27:
**end while**



#### 4.1.2. Offloading Task Set

After step 1, the goal of this step was to determine the computational offloading decision for the task with deadlines. In this paper, we use the back-pressure algorithm to get the decision. Specifically, for the queue of tasks in set *S*, we define $W_{d}^{i}\left(t\right)$ as the length difference of queues between the local queue and the edge queue in the time slot *t*, expressed as

(13) $$W_{d}^{i}\left(t\right)=Q_{l}^{i}\left(t\right)-H_{m}^{i}\left(t\right).$$

Instead of making the offloading decisions directly based on local queue information or the random arrival rate of tasks, the back-pressure algorithm [22] is used as an effective method to reduce the delay. Then we define

(14) $$i^{*}=\arg\max_{i\in S}W_{d}^{i}\left(t\right),$$

where $i^{*}\in S^{*}$ is the task to offload to the edge cloud for computing and $S^{*}$ is the set of offloaded task. In step 2, the final offloading decision of the task and the edge processor *m* to which it will be offloaded can be determined. We can get the number of tasks that can be offloaded based on the offloading decision.

After computing the delay of the task, this step also considers the backlog of tasks of queues in the system to make a more appropriate decision for the task, while also minimizing the computational delay.

#### 4.1.3. Feasibility Check

In the end, for all the tasks to be executed in this system, we define their completion time as the sum of the execution delay and the waiting delay on the local device or edge processor. For the task *i*, we denote its waiting delay on the local device *l* and edge processor *m* in slot *t* as $wait_{l}^{i}\left(t\right)$ and $wait_{m}^{i}\left(t\right)$. It is computed by dividing the queue length of the task *i* by its corresponding execution rate. Then, we check whether the completion time of task meets its deadline $F_{i}$. If so, we execute the corresponding task offloading decision to compute locally or offload to the edge cloud, otherwise the policy cannot make a feasible offloading decision, at which point we execute the fallback option. The fallback option refers to offloading the task directly to the remote cloud for execution. Although the remote cloud is expensive to execute, it has high-speed access and infinite computing power to ensure that the task deadline is met, so this option is feasible.

### 4.2. Performance of the BMDCO Algorithm

**Theorem** **1.** *Queues stability.*

We assume the performance of this algorithm is proportional to the optimal solution by $\frac{1}{1+\theta }$, the corresponding capacity region will be reduced by $\frac{1}{1+\theta }\lambda_{m}I$, where $\lambda_{m}$ is the maximum arrival rate for all i∈N,l∈L. Then, the average queue lengths satisfy

(15) $$\begin{array}{cc} \lim_{T\to \infty } & \frac{1}{T}\sum_{t=1}^{T}\sum_{m=1}^{M}\sum_{i=1}^{N}H_{m}^{i}\left(t\right)\\ \; & \leq \lim_{T\to \infty }\frac{1}{T}\sum_{t=1}^{T}\sum_{l=1}^{L}\sum_{i=1}^{N}Q_{l}^{i}\left(t\right)\\ \; & \leq \frac{\left(1+\theta \right)B_{1}}{\varepsilon -\frac{1}{1+\theta }\lambda_{m}}, \end{array}$$

where ε is a small positive constant. Theorem 1 represents the stability of queues in the system, and the proof of it is shown in Appendix B.

The instability of queues will increase the latency of tasks in computational offloading, and may result in the failure of the computing task with low delay requirements. Therefore, the queues stability is analyzed by deducing that all local queues and edge queues in the queue system are less than a certain value, i.e., they are all have upper bounds, which proves the stability of the BMDCO algorithm. In addition, because of the coupling relationship between the local queue and the edge queue, the departure of a task on a local device queue equals the arrival of the task on an edge queue. So, in Theorem 1, the queue length of the edge queue is not greater than that of the local device queue.

## 5. Numerical Results

In this section, We evaluate the performance of the BMDCO algorithm through numerical simulation. We conducted the simulation experiment on a desktop with MATLAB R2016b, 8 GB RAM, Intel i5 3.20 GHz CPU, and Windows 10 operating system. In our simulation, the smart devices are randomly distributed and each device has its available edge processor, which is evenly distributed, and the remote public cloud is 10 km away from it. We assume that the random arrival of tasks follows the Poisson distribution, and the average arrival rate is the same for all devices and processors, denoted as λ. We consider the arrival of 100 computing tasks on each device and set the task size is 500 bits, and the number of CPU cycles required to compute a task is 50 Mcycles. We set the task completion deadline $F_{i}$ is 5 s. The CPU frequency of local devices are 0.5, 1.0, 1.2, 1.5, 1.8, 2.0, 2.5, and 3.0 GHz, and assume that edge processors all have the same computation capacity for easy comparison. To make the system realistic, we set the scaling factor α(t)=0.95. The noise power is σ=−174 dBM/Hz [20] and the bandwidth is 5 MHz. The channel gain is 0.1 and the transmission power is 4 mW. The maximum transmission rate of the channel is defined as $v_{lm}^{max}\left(t\right)=10^{10}$ bit/s. We define that the initial value of tasks on the local queue and the edge queue is the same, which is represented by *Q*, and set $Q_{max}\left(t\right)$ to be a sufficiently large value. The remaining variables are given in the following detailed analysis.

### 5.1. Performance Analysis

**Figure 2:**
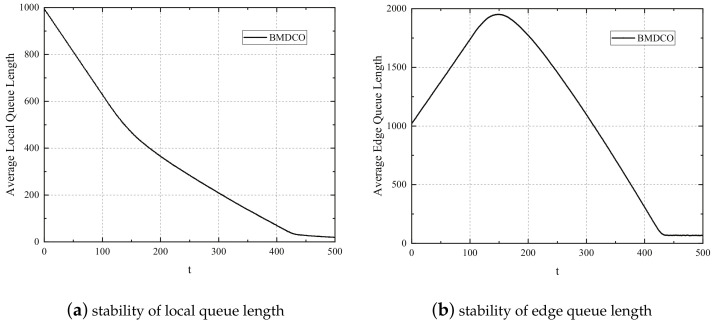
Stability of queue length.

The stability of the BMDCO algorithm is proved by deducing the upper bound of all queues in this queue system, as shown in Figure 2, which includes the stability of local queues and edge queues. In Figure 2, we consider 100 tasks and the average task arrival rate obeys the Poisson distribution of parameter λ=6. We set L=20, M=3, τ=60 ms, and the computation capacity of the edge processors is 35 GHz. The frequency of local device is selected from the given frequency value. The initial value of task queues Q=1000 and the number of iterations is T=500.

In Figure 2a, the result shows the stability of local task queue. When *t* is from 0 to 430, the average queue backlog of local devices shows a rapid and smooth decline, and there is a little flattening at t=150. The reason for this phenomenon is that, when the task on the local device arrives, the local device determines the execution location of these tasks according to the offloading decision. At this time, the local device and the edge processor have sufficient computing resources, which leads to the rapid reduction of the task backlog in the local queue; however, when the local device needs to offload a large number of tasks to the edge cloud, there is a small flattening due to the capacity limit of the transmission channel or the large number of tasks already existing on the edge queue. The queue backlog starts to approach around 28 after t=430 and fluctuates within a certain range, which confirms the stability of local queue.

Figure 2b demonstrates the stability of the edge task queue in this queue system. The graph presents a trend of first increasing and then decreasing. When *t* is from 0 to 150, a large number of tasks on the local device are offloaded to the edge cloud, and it also has many tasks to be calculated, so the backlog of tasks in the edge queue is increasing. After t=150, the task backlog of edge queue begins to show a downward trend. This is because the edge processor has enough computing resources to reach a balance with the local queue after computing a large number of tasks offloaded from the local, which causes a reduction in the backlog of tasks in this queue. Finally, the queue length tends to be flat within certain limits when t=430, which verifies the edge queue stability.

### 5.2. Performance Comparison

We compare the performance of the BMDCO algorithm with the following five alternatives:

- Only Local Execution (LE): Tasks are only executed on the local device, and only local state information is considered.
- Only Edge Cloud Execution (ECE): The algorithm offloads all tasks to the edge processor for computing and determines the offloading decision by considering the information of transmission channel and edge cloud.
- Random Computational Offloading (RCO): This algorithm uses the queue method, and the task offloading matrix is randomly generated to determine where the task is performed.
- Back-pressure Algorithm-based Computational Offloading (BPCO) [45]: This method considers a queuing system, and determines the task offloading decision by using the back-pressure algorithm to calculate the task backlog difference between the local queue and the edge queue.
- Closed-form Delay-optimal Computational Offloading (CDCO) [20]: This strategy takes into account the queue model and determines the offloading decision of tasks by minimizing the average energy cost.

**Figure 3:**
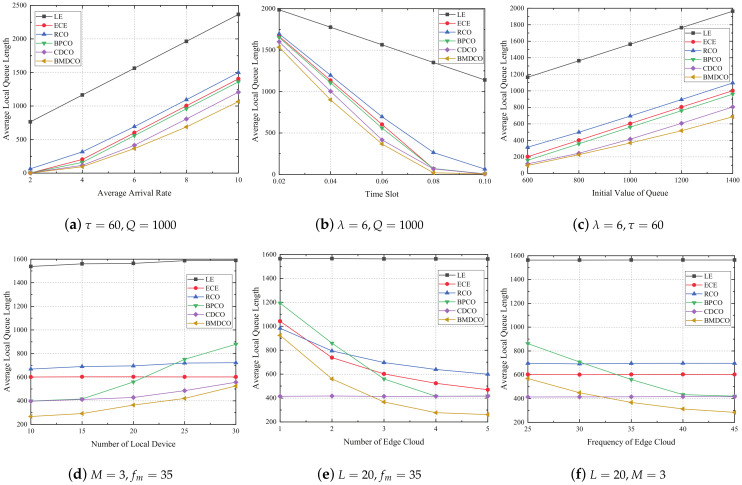
Performance comparison graph.

In this section, we mainly compare the average local queue backlog between the proposed BMDCO algorithm and the other five methods under the following situations: (1) Average Arrival Rate, (2) Time Slot, (3) Initial Value of Queue, (4) Number of Local Device, (5) Number of Edge Cloud, and (6) Frequency of Edge Cloud. The performance comparison is shown in Figure 3. The results show that the performance of BMDCO is better than the other alternatives, because we not only consider the computational delay but also consider the task backlog difference between local queue and edge queue. The detailed performance description for each case is described as follows.

In Figure 3a–c, we consider that there are 100 tasks per local device and the edge processor has a CPU frequency of 35 GHz; we set L=20 and M=3. To facilitate numerical comparison, we take the number of iterations T=200. These three diagrams show the change of the local queue backlog as the task arrival rate, the length of the time slot, and the initial value of the queue change. In Figure 3a, we set τ=60 ms and Q=1000. It is observed that, as the task arrival rate increases, the queue length of the BMDCO algorithm also increases due to constraints such as transmission channels, but when λ is larger, it is obviously better than other methods. Figure 3b describes the change of the average local queue backlog as the time slot changes when λ=6 and Q=1000. With the increase of time slot τ, this method calculates the most tasks, so the local queue backlog is the smallest. Figure 3c presents the change when the initial value of the queue is changed. When other conditions are the same, increase the initial value of all queues in the system, and we can find that the growth of the BMDCO algorithm is the slowest.

Figure 3d–f illustrate the changes of average local task backlog in the case of different number of local devices, different number of edge clouds, and different processing capacities of edge processors. When the definitions of other variables are the same, Figure 3d investigates the relationship between the number of local devices and the backlog of local queues. This figure shows that the ECE method has the same average local queue length because tasks are only processed on the edge cloud. With the increase of local equipment, the average queue backlog of the proposed BMDCO algorithm is smaller than the other methods. Figure 3e depicts the performance of local queue backlog under different edge cloud numbers. It shows that the CDCO algorithm is almost unaffected, because the goal of it is to minimize system energy consumption. It only calculates the energy consumption of the local and transmission process, and the energy consumption of the edge cloud processor is not considered. The LE algorithm is also unaffected because it only computes locally. From this graph, we observe that BMDCO algorithm is superior to other methods. In Figure 3f, we demonstrate the changes in the task backlog of local queue under different edge cloud processing capabilities. It can be seen that the performance of LE, RCO, and CDCO is not directly related to the change of edge cloud computing capabilities. Due to a certain transmission capacity, the performance of the ECE method is less affected. Compared to the BPCO algorithm, the proposed algorithm can reach better performance.

### 5.3. Summary

We study the stability of the algorithm and evaluate its performance. In the performance analysis, it can be verified that the queues in the proposed BMDCO method are all upper bound by adjusting the number of iterations. The specific analysis is performed through the local queue and the edge queue, respectively. In the second subsection, we respectively compare the performance of this algorithm with other five alternatives in terms of time slot, average arrival rate of tasks, size of initial queue value, number of local devices, number of edge cloud processors, and the frequency of edge cloud processing. It can be seen from the simulation results that the average local queue backlog of the BMDCO algorithm is smaller than other methods in these aspects, which verifies that this algorithm can effectively reduce the computational delay of tasks and improve the performance of local intelligent devices.

## 6. Conclusions

The over-concentration of services in smart homes will lead to the decrease of its operating efficiency, and the distributed processing of its computing can effectively meet the needs of users. Therefore, we use computational offloading techniques in edge computing to reduce the computational delay and improve the response rate. In this paper, we studied the problem of offloading decision-making in a busy queue system in which tasks arrive randomly. Then, we used the Lyapunov optimization method to minimize the average queue length of all queues in the system and realize the stability of the queues. Furthermore, we presented the BMDCO algorithm, which can obtain the task decision and offloading number while minimizing the computational delay of the sensitive task with deadlines. At the same time, we proved the stability of the BMDCO algorithm through theoretical analysis. The simulation results verified the boundedness of the queue and showed that the proposed strategy has better performance than other conventional methods and can effectively reduce user delay. In next work, we will also consider the energy consumption of the task as one of the major constraints in the problem formulation.

## Data Availability

The study did not report any data.

## Appendix A. Lyapunov Drift Optimization Problem

According to Equation (7) and *Lemma 7* in [22], we get an inequality about the local device queue $Q_{l}^{i}\left(t\right)$ as follows:

(A1) $$\begin{array}{c} \frac{1}{2}\left[{\left(Q_{l}^{i}\left(t+1\right)\right)}^{2}-{\left(Q_{l}^{i}\left(t\right)\right)}^{2}\right]\leq B_{1}-Q_{l}^{i}\left(t\right)\left(v_{l}^{i}\left(t\right)+\sum_{m=1}^{M}y_{lm}^{i}\left(t\right)v_{lm}^{i}\left(t\right)-A_{l}^{i}\left(t\right)\right), \end{array}$$

where $B_{1}=\frac{1}{2}\left[{\left(v_{l}^{i}\left(t\right)+\sum_{m=1}^{M}y_{lm}^{i}\left(t\right)v_{lm}^{i}\left(t\right)\right)}^{2}+{\left(A_{l}^{i}\left(t\right)\right)}^{2}\right]$. In the same way, from Equation (8), we can obtain

(A2) $$\begin{array}{c} \frac{1}{2}\left[{\left(H_{m}^{i}\left(t+1\right)\right)}^{2}-{\left(H_{m}^{i}\left(t\right)\right)}^{2}\right]\leq B_{2}-H_{m}^{i}\left(t\right)\left(v_{m}^{i}\left(t\right)-\sum_{l=1}^{L}y_{lm}^{i}\left(t\right)v_{lm}^{i}\left(t\right)-A_{m}^{i}\left(t\right)\right), \end{array}$$

where $B_{2}=\frac{1}{2}\left[{\left(v_{m}^{i}\left(t\right)\right)}^{2}+{\left(\sum_{l=1}^{L}y_{lm}^{i}\left(t\right)v_{lm}^{i}\left(t\right)+A_{m}^{i}\left(t\right)\right)}^{2}\right]$.

According to the above two conclusion, we sum Equations (A1) and (A2) and take the expectation over i∈{1,…,N}, l∈{1,…,L} and m∈{1,…,M}, which can obtain the Lyapunov drift as follows:

(A3) $$\begin{array}{cc} \Delta (t)\leq B & -E\left[\sum_{l=1}^{L}\sum_{i=1}^{N}Q_{l}^{i}\left(t\right)\left(v_{l}^{i}\left(t\right)+\sum_{m=1}^{M}y_{lm}^{i}\left(t\right)v_{lm}^{i}\left(t\right)-A_{l}^{i}\left(t\right)\right)\right]\\ & -E\left[\sum_{m=1}^{M}\sum_{i=1}^{N}H_{m}^{i}\left(t\right)\left(v_{m}^{i}\left(t\right)-\sum_{l=1}^{L}y_{lm}^{i}\left(t\right)v_{lm}^{i}\left(t\right)-A_{m}^{i}\left(t\right)\right)\right], \end{array}$$

in which *B* is a finite constant and $B=\sum_{l=1}^{L}\sum_{i=1}^{N}E\left[B_{1}\right]+\sum_{m=1}^{M}\sum_{i=1}^{N}E\left[B_{2}\right]$. Minimizing the Lyapunov drift function Δ(t) is equivalent to maximizing Equation (12).

## Appendix B. Proof of Theorem 1

Let $y_{lm}^{i*}\left(t\right),v_{l}^{i*}\left(t\right),v_{lm}^{i*}\left(t\right)$ represent the optimal solution of Equation (12). According to Equation (A1), we obtain

(A4) $$\begin{array}{c} B_{1}+Q_{l}^{i}\left(t\right)A_{l}^{i}\left(t\right)\leq Q_{l}^{i}\left(t\right)\left(v_{l}^{i*}\left(t\right)+\sum_{m=1}^{M}y_{lm}^{i*}\left(t\right)v_{lm}^{i*}\left(t\right)\right). \end{array}$$

Noted that if every average arrival rates satisfy Equation (A4), all average arrival rates satisfy the queue stability region R. For any task arrival rate in R, the average execution rate should not be less than $\lambda_{l}^{i}$ plus ε. So we can obtain the Lyapunov drift as:

(A5) $$\begin{array}{cc} \; & E\left[\sum_{l=1}^{L}\sum_{i=1}^{N}{\left(Q_{l}^{i}\left(t+1\right)\right)}^{2}\right]-E\left[\sum_{l=1}^{L}\sum_{i=1}^{N}{\left(Q_{l}^{i}\left(t\right)\right)}^{2}\right]\\ & \leq 2B_{1}+\sum_{l=1}^{L}\sum_{i=1}^{N}2E\left[Q_{l}^{i}\left(t\right)\right]\lambda_{l}^{i}-\sum_{l=1}^{L}\sum_{i=1}^{N}2E\left[Q_{l}^{i}\left(t\right)\right]\left(\lambda_{l}^{i}+\varepsilon \right)\\ & \leq 2B_{1}-2\varepsilon \sum_{l=1}^{L}\sum_{i=1}^{N}E\left[Q_{l}^{i}\left(t\right)\right]. \end{array}$$

Then, summing over i∈{1,…,N} and taking the limit of *T*, we have

(A6) $$\begin{array}{c} \lim_{T\to \infty }\frac{1}{T}\sum_{t=1}^{T}\sum_{l=1}^{L}\sum_{i=1}^{N}E\left[Q_{l}^{i}\left(t\right)\right]\leq \frac{B_{1}}{\varepsilon }. \end{array}$$

If the performance of the algorithm proposed in the paper is proportional to the optimal solution by $\frac{1}{1+\theta }$, then

(A7) $$\begin{array}{cc} \sum_{l=1}^{L}\sum_{i=1}^{N}Q_{l}^{i}\left(t\right) & \left(v_{l}^{i*}\left(t\right)+\sum_{m=1}^{M}y_{lm}^{i*}\left(t\right)v_{lm}^{i*}\left(t\right)\right)\\ & \leq \left(1+\theta \right)\sum_{l=1}^{L}\sum_{i=1}^{N}Q_{l}^{i}\left(t\right)\left(v_{l}^{i}\left(t\right)+\sum_{m=1}^{M}y_{lm}^{i}\left(t\right)v_{lm}^{i}\left(t\right)\right). \end{array}$$

Substituting Equation (A7) into Equation (A4) and taking the expectation, we have

(A8) $$\begin{array}{c} \frac{B_{1}}{1+\theta }+\frac{\sum_{l=1}^{L}\sum_{i=1}^{N}E\left[Q_{l}^{i}\left(t\right)\right]\lambda_{l}^{i}}{1+\theta }\leq \sum_{l=1}^{L}\sum_{i=1}^{N}Q_{l}^{i}\left(t\right)\left(v_{l}^{i}\left(t\right)+\sum_{m=1}^{M}y_{lm}^{i}\left(t\right)v_{lm}^{i}\left(t\right)\right). \end{array}$$

Due to the capacity region of the proposed algorithm reduced by $\frac{1}{1+\theta }\lambda_{m}$, that is $R^{{}^{\prime }}=R-\frac{1}{1+\theta }\lambda_{m}I$, and the parameter $B_{1}^{{}^{\prime }}=\left(1+\theta \right)B_{1}$. Hence, the average queue length of the local queue should satisfy

(A9) $$\begin{array}{c} \lim_{T\to \infty }\frac{1}{T}\sum_{t=1}^{T}\sum_{l=1}^{L}\sum_{i=1}^{N}E\left[Q_{l}^{i}\left(t\right)\right]\leq \frac{\left(1+\theta \right)B_{1}}{\varepsilon -\frac{1}{1+\theta }\lambda_{m}}. \end{array}$$

In this queue system, due to the coupling between the local device queue and the edge processor queue, so the number of tasks in the local device queue is no less than the edge queue. Therefore, we obtain

(A10) $$\begin{array}{c} E\left[\sum_{t=1}^{T}\sum_{m=1}^{M}\sum_{i=1}^{N}H_{m}^{i}\left(t\right)\right]\leq E\left[\sum_{t=1}^{T}\sum_{l=1}^{L}\sum_{i=1}^{N}Q_{l}^{i}\left(t\right)\right]. \end{array}$$

From Equations (A9) and (A10), we get the Theorem 1, i.e.,

(A11) $$\begin{array}{c} \lim_{T\to \infty }\frac{1}{T}\sum_{t=1}^{T}\sum_{m=1}^{M}\sum_{i=1}^{N}E\left[H_{m}^{i}\left(t\right)\right]\leq \lim_{T\to \infty }\frac{1}{T}\sum_{t=1}^{T}\sum_{l=1}^{L}\sum_{i=1}^{N}E\left[Q_{l}^{i}\left(t\right)\right]\leq \frac{\left(1+\theta \right)B_{1}}{\varepsilon -\frac{1}{1+\theta }\lambda_{m}}. \end{array}$$
